# Headspace, a youth integrated care model: The relation between users satisfaction, clinical and demographic characteristics and service utilization

**DOI:** 10.1192/j.eurpsy.2022.1071

**Published:** 2022-09-01

**Authors:** G. Hoter Ishay, D. Roe

**Affiliations:** University of Haifa, Department Of Community Mental Health, Haifa, Israel

**Keywords:** young people, service satisfaction, treatment gap

## Abstract

**Introduction:**

Youth integrated care services were developed to overcome common barriers to mental health treatment. Satisfaction is key for services utilization and engagement.

**Objectives:**

To study users satisfaction with youth integrated care service, “Headspace”, throughout the course of treatment and its correlation with clinical and demographic characteristics and service utilization.

**Methods:**

A sample of 112 participants ranging between ages 12-25 who attended the Headspace clinic between March 2016 and June 2018 were assessed in the middle (after 7 sessions) and end of treatment (n=71).

**Results:**

Participants expressed high levels of satisfaction across all service aspects at the middle and end of treatment. The highest rate of satisfaction was with the centre’s staff and the lowest with personal outcomes. A repeated measures ANOVA analysis revealed that only satisfaction with personal outcomes improved significantly over time Length of wait to begin treatment and parental engagement y were negatively correlated with youth satisfaction.

**Conclusions:**

Satisfaction rates of Headspace among youth are high from the start and with their outcomes increase over time. Youth satisfaction with the staff’s attitude and approach and satisfaction with accessibility suggest the service achievement in addressing barriers of help seeking in youth.

**Disclosure:**

No significant relationships.

